# Early-Pregnancy Dydrogesterone Supplementation Mimicking Luteal-Phase Support in ART Patients Did Not Provoke Major Reproductive Disorders in Pregnant Mice and Their Progeny

**DOI:** 10.3390/ijms22105403

**Published:** 2021-05-20

**Authors:** Laura Jeschke, Clarisa Guillermina Santamaria, Nicole Meyer, Ana Claudia Zenclussen, Julia Bartley, Anne Schumacher

**Affiliations:** 1 Experimental Obstetrics and Gynecology, Medical Faculty, Otto-von-Guericke University, 39108 Magdeburg, Germany; laura.jeschke@st.ovgu.de (L.J.); clarisa-guillermina.santamaria@ufz.de (C.G.S.); nicole.meyer@ufz.de (N.M.); ana.zenclussen@ufz.de (A.C.Z.); 2 UFZ—Helmholtz Centre for Environmental Research Leipzig-Halle, Department of Environmental Immunology, 04318 Leipzig, Germany; 3 Reproductive Medicine and Gynecological Endocrinology, University Women’s Clinic, Medical Faculty, Otto-von-Guericke University, 39108 Magdeburg, Germany; julia.bartley@med.ovgu.de

**Keywords:** dydrogesterone, early pregnancy, artificial reproductive techniques, luteal-phase support, safety, tolerability, progeny, reproductive disorders

## Abstract

Progestogens are frequently administered during early pregnancy to patients undergoing assisted reproductive techniques (ART) to overcome progesterone deficits following ART procedures. Orally administered dydrogesterone (DG) shows equal efficacy to other progestogens with a higher level of patient compliance. However, potential harmful effects of DG on critical pregnancy processes and on the health of the progeny are not yet completely ruled out. We treated pregnant mice with DG in the mode, duration, and doses comparable to ART patients. Subsequently, we studied DG effects on embryo implantation, placental and fetal growth, fetal-maternal circulation, fetal survival, and the uterine immune status. After birth of in utero DG-exposed progeny, we assessed their sex ratios, weight gain, and reproductive performance. Early-pregnancy DG administration did not interfere with placental and fetal development, fetal-maternal circulation, or fetal survival, and provoked only minor changes in the uterine immune compartment. DG-exposed offspring grew normally, were fertile, and showed no reproductive abnormalities with the exception of an altered spermiogram in male progeny. Notably, DG shifted the sex ratio in favor of female progeny. Even though our data may be reassuring for the use of DG in ART patients, the detrimental effects on spermatogenesis in mice warrants further investigations and may be a reason for caution for routine DG supplementation in early pregnancy.

## 1. Introduction

Progesterone (P4) fulfills pivotal functions in early pregnancy stages such as preparing the endometrium for proper embryo implantation, regulating uterine contractility, and modulating maternal immune responses toward fetal antigens [1]. Consequently, a loss of P4 in this critical phase often ends up in embryo implantation failure and/or miscarriage. Under spontaneous pregnancy conditions, P4 is first produced by the corpus luteum and later on by the placenta. Controlled ovarian stimulation (COS) in assisted reproductive techniques (ART) often induces luteal insufficiency and thus provokes a P4 deficit. To overcome P4 scarcity, patients undergoing COS are routinely supplemented with exogenous progestogens through different routes, including oral, intramuscular, vaginal, and subcutaneous application forms [2]. P4 supplementation commonly starts on the day of or the day after egg collection, and is continued at least until the first positive pregnancy test [2]. Additionally, progestogens are administered to patients with threatened or recurrent miscarriages during the first trimester to support successful pregnancy progression; however, the benefit of this treatment is controversially discussed [3].

Although the use of micronized P4 supplementation is effective and safe, the need of a vaginal or subcutaneous application is inconvenient for the majority of patients. Therefore, the use of dydrogesterone (DG), a biologically active metabolite of P4 with good oral bioavailability, has become an alternative to micronized P4 in clinical practice with a high level of patient compliance [2]. DG, as a synthetic progestogen, has to be supplemented until the 12th week of pregnancy, resembling the complete first trimester of pregnancy. Two recent meta-analyses provided evidence that DG administration as luteal-phase support in ART patients shows at least similar or even higher efficacy compared to vaginal progestogens with regard to clinical pregnancy rates and ongoing pregnancy/live birth rates [4,5]. Moreover, there seem to be no relevant differences in miscarriage rates and other maternal and fetal/newborn adverse effects between both application forms [5]. Nevertheless, it has to be admitted that due to the requirement for a continuous supplementation of DG until the luteal-placental shift [6], there are concerns about the safety of DG during the sensible first-trimester pregnancy phase that may not only affect fetal organogenesis, but may also interfere with later health issues in the newborn, growing child, or adult, and may even generate intergenerational effects [3]. In particular, as sex-specific differentiation, development, and function of the reproductive system are largely dependent on steroid hormones [7], alterations in fetal hormone exposure can have lifelong effects [8]. Moreover, DG is currently not a licensed drug in the UK or USA [3], and there is evidence for potential harm from this synthetic progestogen [9]. To provide a recommendation for the further use of DG during early pregnancy and to shed more light on the mechanisms underlying DG action, our current study addressed three major questions: (1) whether preimplantation DG treatment affects the uterine environment and thus embryo implantation; (2) whether early-pregnancy (first trimester) DG treatment influences fetal and placental growth, maternal-fetal blood circulation, or fetal survival; and (3) whether early-pregnancy (first trimester) DG treatment impacts on the reproductive performance of the progeny. We performed our study in the mouse system using DG doses, mode, and length of administration comparable to ART patients.

Our findings indicated no major effects of DG on pregnancy-related processes such as embryo implantation, placentation, maternal-fetal circulation, fetal growth, and hormonal immune modulation as compared to vehicle-treated controls. Progeny of DG-treated dams grew normally, were fertile, and showed no reproductive abnormalities, with the exception of altered sperm parameters in male offspring. Interestingly, DG administration shifted the female-to-male progeny ratio in favor of the females.

However, a potential harmful effect of DG on spermatogenesis of the F1 generation has to be further validated, and our current findings do not rule out the use of DG as luteal-phase support in ART patients in general.

## 2. Results

Preimplantation DG treatment induced minor changes in the uterine composition of immune and implantation-related markers, but did not affect embryo implantation at gestation day (GD)5.

ART patients frequently receive oral DG supplementation even before the embryo is transferred into the uterine cavity. Consequently, DG may provoke alterations in the cellular and molecular composition of the uterine environment that ultimately affect the capability of the embryo to successfully implant into the maternal endometrium. To address this issue, we examined the mRNA expression of several pro- and anti-inflammatory immune factors and pro-implantation molecules, as well as the proportions of adaptive immune cell subsets in the uterine tissue after DG supplementation in the preimplantation period. We did not observe significant changes for the majority of factors and molecules that were studied at implantation (Figure S1). We only observed changes for *IL-1b*, *IL-10*, and *FOXP3*, the mRNA expression of which were significantly reduced after supplementation with 2 mg/kg DG (Figure S1). Likewise, uterine proportions of B and T cell subsets were not significantly affected, with the exception of CD19+IL-10+ B cells and CD4+RORgt+ T cells that were significantly augmented after treatment with 6 mg/kg DG when compared to 2 mg/kg DG (Table 1). Study of the potential effects of DG treatment on embryo implantation revealed comparable numbers of implantations among the groups (control group: 64 implantations out of 8 dams; 2 mg/kg DG group: 74 implantations out of 9 dams; 6 mg/kg DG group: 67 implantations out of 8 dams). These observations suggest that DG supplementation during very early pregnancy stages induced only minor changes in the uterine environment, with no consequences for the ability of the embryo to implant.

Early-pregnancy DG treatment provoked a short-term increase in implantation sizes at GD10, but did not influence fetal and placental growth or fetal survival at GD12.

**Figure 1:**
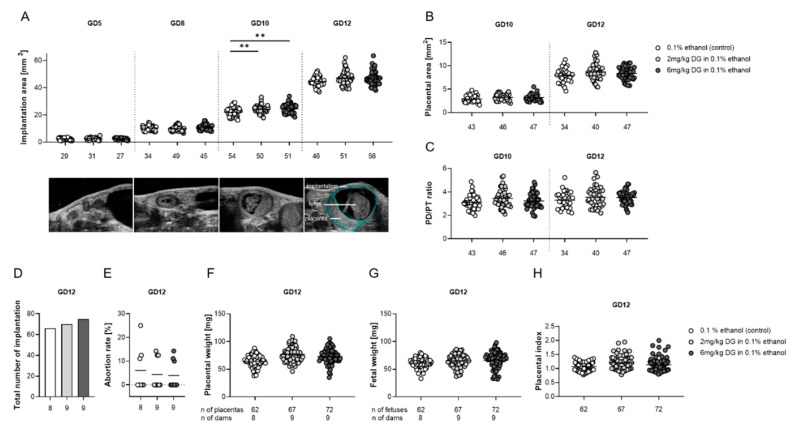
Dydrogesterone (DG) treatment induced a short-term increase in implantation sizes at GD10, but did not alter placental and fetal parameters at GD12. Pregnant females were injected with 0.1% ethanol (*n* = 8), 2 mg/kg DG (*n* = 9), or 6 mg/kg DG (*n* = 9) from GD0–GD10. The graphics show (**A**) implantation areas, (**B**) placental areas, (**C**) ratio of placental diameter to placental thickness, (**D**) total number of implantation, (**E**) abortion rate, (**F**) placental weight, (**G**) fetal weight, and (**H**) placental index. Data are shown as means plus S.D. and were analyzed for statistical differences either by using one-way ANOVA followed by Tukey’s multiple comparison test or by applying a mixed linear model using the final test principal. ** *p* < 0.01 vs. control.

Next, we wondered whether murine DG supplementation corresponding to the first trimester period in pregnant patients would affect fetal and placental development, as well as fetal survival. We followed up implantation sizes from GD5 onward by high-frequency sonography, and found significantly bigger implantation areas in both DG treatment groups compared to the control group at GD10; however, these differences became statistically irrelevant at GD12 (Figure 1A). Representative pictures of whole implantation sites for each GD are displayed next to the graphic. Similarly, ultrasound-derived data of placental areas (Figure 1B), diameters, and thicknesses and ratios of placental diameters-to-thicknesses (PD/PT ratio; Figure 1C) at GD10 and 12 did not reveal significant differences among the groups. After sacrificing the pregnant dams at GD12, we did not observe changes in the total number of implantations or in the abortion rates between DG treatments and controls (Figure 1D,E). Likewise, fetal and placental weights, as well as placental-fetal (placental) indices, were not influenced by both DG doses (Figure 1F–H). Our findings suggested that early-pregnancy DG supplementation does not affect critical pregnancy processes.

Early-pregnancy DG treatment did not alter utero- and fetal-placental blood flow indices from implantation to midgestation.

**Figure 2:**
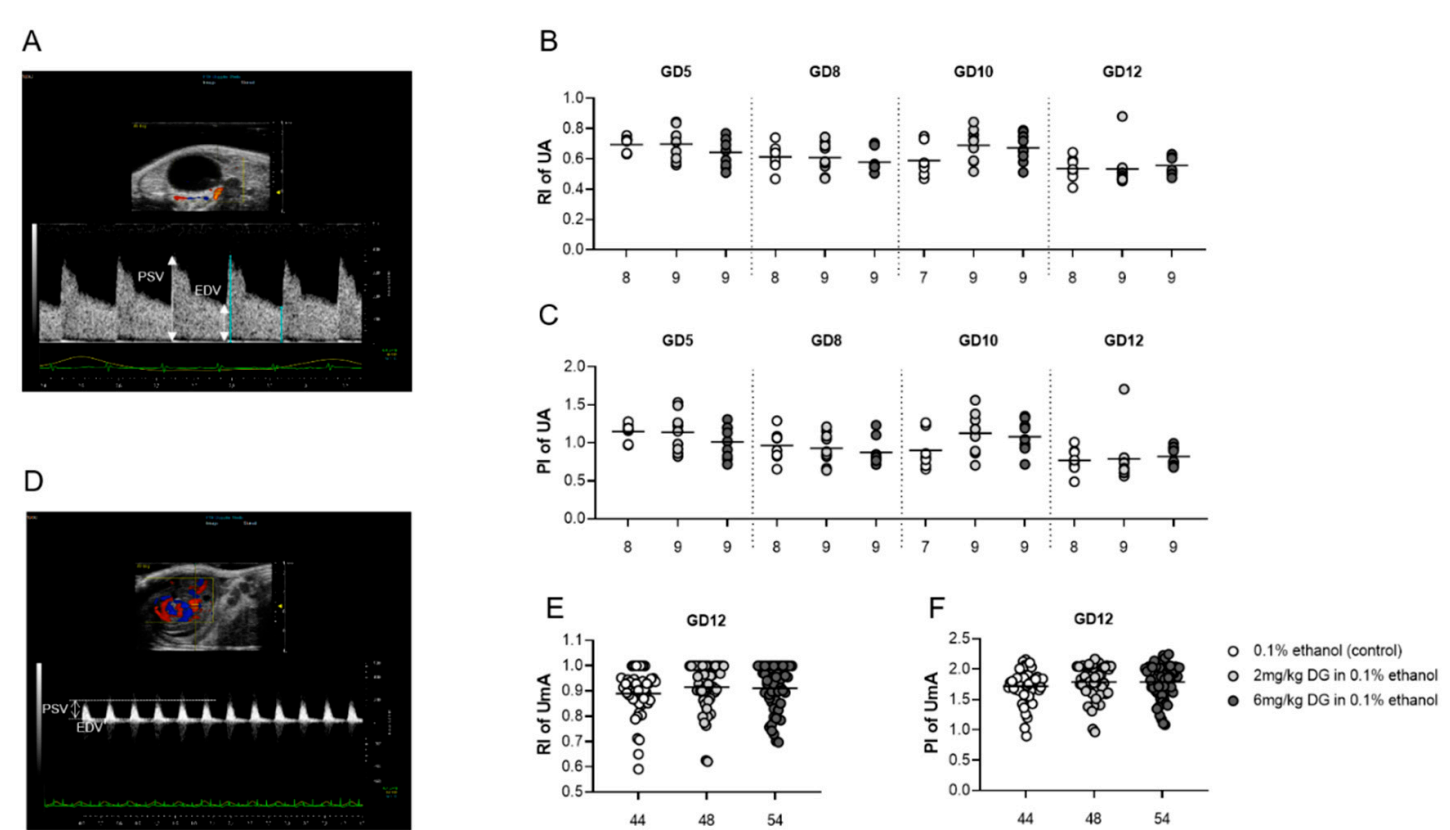
Dydrogesterone (DG) treatment had no influence on maternal- and fetal-placental blood-flow parameters in the postimplantation period. Pregnant females were injected with 0.1% ethanol (*n* = 8), 2 mg/kg DG (*n* = 9), or 6 mg/kg DG (*n* = 9) from GD0–GD10. Graphics and figures show (**A**) location of the uterine artery and corresponding Doppler flow diagram, (**B**) resistance index of the uterine artery, (**C**) pulsatility index of the uterine artery, (**D**) location of the umbilical artery and corresponding Doppler flow diagram, (**E**) resistance index of the umbilical artery, and (**F**) pulsatility index of the umbilical artery. Data are shown as means plus S.D. and were analyzed for statistical differences by using one-way ANOVA followed by Tukey’s multiple comparison test or by applying a mixed linear model using the final test principal. RI—resistance index, PI –pulsatility index, PSV—peak systolic velocity, EDV—end-diastolic velocity, UA—uterine artery, UmA—umbilical artery.

Uterine and umbilical Doppler sonography allows analyses of the utero- and fetal-placental circulation, and is therefore an adequate tool to identify insufficient fetal blood supply, indicated by increased vascular impedance that in turn is reflected by increased resistance (RI) and pulsatility (PI) indices. We performed Doppler measurements in the maternal uterine arteries (UA) at GD5, 8, 10, and 12, as well as in the umbilical arteries (UmA) at GD12. Figure 2A,D display the location of the UA and UmA in situ, respectively, as well as a representative Doppler flow diagram. Application of both DG doses did not result in changes of RI and PI in the maternal and fetal-placental circulation (Figure 2B,C,E,F). Our data did not support DG-mediated changes in the establishment and maintenance of the maternal-fetal blood circulation.

Early-pregnancy DG treatment resulted in decreased uterine *IL-10* mRNA expression and diminished uterine proportions of IL-10- and PD-1-expressing CD4+ T cells at GD12.

In order to identify alterations in the uterine immune composition upon early-pregnancy DG treatment, we analyzed the mRNA expression of various pro- and anti-inflammatory immune factors, as well as the proportions of B and T cell subsets at GD12. Our analyses showed that the lower dose of DG significantly decreased the *IL-10* mRNA expression in the uterine tissue as compared to the control group (Figure S2F). Notably, proportions of IL-10- and PD-1-expressing CD4+ T cells were also significantly reduced after application of 2 mg/kg DG when compared to control mice (Table 2). All other factors and cell subsets studied were comparable among the groups (Figure S2 and Table 2). Based on these observations, we state that DG supplementation during early pregnancy stages had no profound effects on the uterine immune status at midgestation.

DG treatment of pregnant dams shifted the female-to-male ratio of their progeny, provoked a short-term weight gain in female offspring, and altered sperm parameters in male offspring.

**Figure 3:**
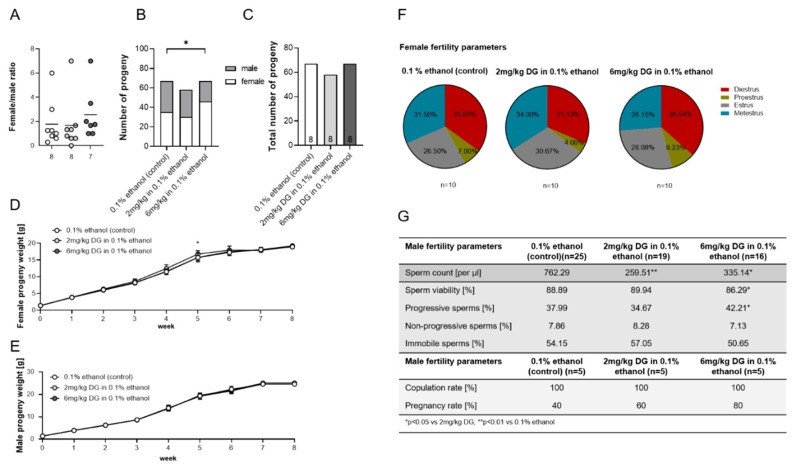
Dydrogesterone (DG) treatment of pregnant dams shifted progeny’s sex ratio, affected the weight of female progeny, and impaired sperm quality and quantity of male progeny. Pregnant dams were injected with 0.1% ethanol (*n* = 8), 2 mg/kg DG (*n* = 8), or 6 mg/kg DG (*n* = 8) from GD0–GD10. Graphics and table show (**A**) ratio of female to male progeny, (**B**) number of all progeny, (**C**) total number of all progeny, (**D**) female progeny weight, (**E**) male progeny weight, (**F**) female fertility parameters, and (**G**) male fertility parameters. Data are shown as means plus S.D. and were analyzed for statistical differences by using one-way ANOVA followed by Tukey’s multiple comparison test, by applying a mixed linear model using the final test principal, or by using the chi square test. * *p* < 0.05 vs. 6 mg/kg DG and control (body weight).

After we focused our analyses on pregnancy itself, we were interested in figuring out whether progeny of DG-exposed dams would show any modifications in their ability to gain weight during the first 8 weeks of life and would be as fertile as the progeny of vehicle-exposed dams. Remarkably, we observed a shift in the female-to-male progeny ratio in favor of the females after 6 mg/kg DG (Figure 3A) treatment of the mothers, which resulted in significantly more female progeny in these litters compared to litters from vehicle-treated dams (Figure 3B). This observation was not due to changes in the total numbers of born babies among the groups (Figure 3C). To be sure that the phenotypic determination of the progeny’s sex was identical to the genotypic-defined sex, all progeny were genotyped at the age of 3 weeks. There were no disparities between the phenotype and the genotype of all progeny. Then we followed up the weight gain of the progeny of both sexes separately and found that females born to 2 mg/kg DG-exposed dams were significantly heavier at 5 weeks of age as compared to female progeny of 6 mg/kg DG-exposed and vehicle-exposed dams (Figure 3D). However, female progeny weights were comparable among all groups for every other time point tested (Figure 3D), and this was also true for male progeny (Figure 3E). Further analyses of female fertility parameters at the age of 6–8 weeks revealed that the length of each estrus cycle stage was the same in female progeny of DG-exposed dams when compared to female progeny of vehicle-exposed dams (Figure 3F). Similarly, the number of corpora lutea did not differ among groups. On the contrary, data obtained on sperm numbers, viability, and motility showed that male progeny from dams exposed to 2 mg/kg DG had significant lower total counts of sperms at the age of 6 weeks. Moreover, sperms of male progeny born to dams that were previously exposed to 6 mg/kg were in general less viable, but living sperms showed higher motility (Figure 3G). Based on these results, we assumed an effect on the capability of male progeny from DG-exposed dams to impregnate BALB/c females. Whereas copulation rates were comparable among groups, females mated to male progeny from DG-exposed dams were more likely to become pregnant than females mated to male progeny from vehicle-exposed dams (Figure 3G). Altogether, in utero exposure to DG induced a sex disparity among the progeny and altered the spermiogram of the male progeny, but did not affect the growth development and the capability of both sexes to reproduce themselves.

Early-pregnancy DG treatment of dams did not affect fetal and placental growth, utero- and fetal-placental blood flow indices, or fetal survival in subsequent pregnancies of female progeny.

**Figure 4:**
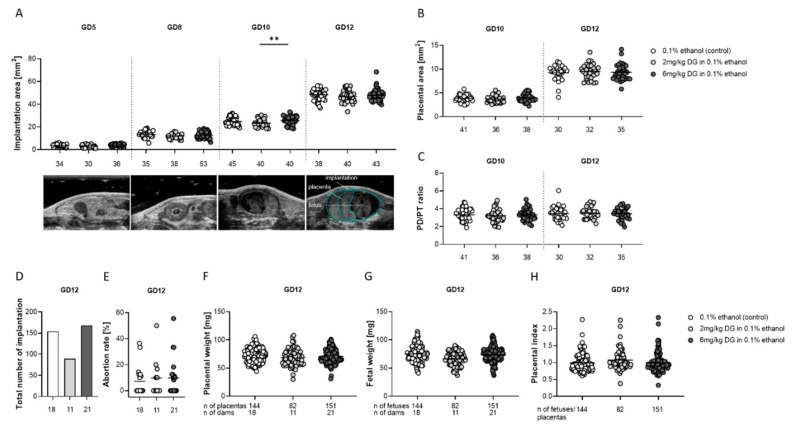
Dydrogesterone (DG) treatment of pregnant dams did not provoke any changes in placental and fetal parameters or in fetal survival in subsequent pregnancies of female progeny. Pregnant dams were injected with 0.1% ethanol (*n* = 18), 2 mg/kg DG (*n* = 11), or 6 mg/kg DG (*n* = 21) from GD0–GD10. The graphics show (**A**) implantation areas, (**B**) placental areas, (**C**) ratio of placental diameter to placental thickness, (**D**) total number of implantation, (**E**) abortion rate, (**F**) placental weight, (**G**) fetal weight, and (**H**) placental index. Data are shown as means plus S.D. and were analyzed for statistical differences either by using one-way ANOVA followed by Tukey’s multiple comparison test or by applying a mixed linear model using the final test principal. ** *p* < 0.01 vs. 2 mg/kg DG.

After we confirmed that male progeny of DG-treated dams were fertile, we sought to investigate whether their female counterparts were able to become pregnant and whether pregnancy would progress normally. We followed up the growth of whole implantation areas, placentas, and fetuses starting at GD5 by high-frequency sonography, and determined pregnancy outcomes as well as fetal and placental weights at GD12. Previous DG treatment of pregnant dams did not influence fetal and placental development or fetal survival in next-generation pregnancies (Figure 4). We found comparable implantation sizes (Figure 4A), placental areas (Figure 4B), diameters and thicknesses, and PD/PT ratios (Figure 4C) in pregnant female progeny of DG-exposed dams when compared to vehicle-exposed dams. At GD10, implantation areas of pregnant female progeny previously exposed to the high DG dose were significantly larger than implantation areas of pregnant female progeny previously exposed to the low DG dose (Figure 4A). After sacrificing the pregnant female progeny at GD12, we observed equal numbers of implantations and similar abortion rates regardless of the treatment of the dams (Figure 4D,E). Likewise, fetal and placental weights, as well as placental-fetal (placental) indices, were not affected by previous DG treatment (Figure 4F–H). Previous in utero exposure to DG had no effects on subsequent pregnancies in female progeny.

**Figure 5:**
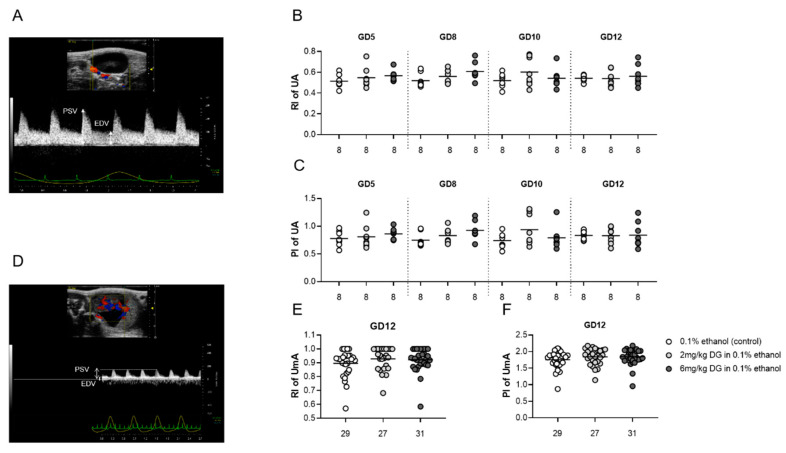
Dydrogesterone (DG) treatment of pregnant dams did not influence maternal- and fetal-placental blood-flow parameters in pregnant female progeny. Pregnant dams were injected with 0.1% ethanol (*n* = 8), 2 mg/kg DG (*n* = 8), or 6 mg/kg DG (*n* = 8) from GD0–GD10. Graphics and figures show (**A**) location of the uterine artery and corresponding Doppler flow diagram, (**B**) resistance index of the uterine artery, (**C**) pulsatility index of the uterine artery, (**D**) location of the umbilical artery and corresponding Doppler flow diagram, (**E**) resistance index of the umbilical artery, and (**F**) pulsatility index of the umbilical artery. Data are shown as means plus S.D. and were analyzed for statistical differences by using one-way ANOVA followed by Tukey’s multiple comparison test or by applying a mixed linear model using the final test principal. RI—resistance index, PI—pulsatility index, PSV—peak systolic velocity, EDV—end-diastolic velocity, UA—uterine artery, UmA—umbilical artery.

Sonographic measurement of blood-flow parameters further revealed no significant changes of RI and PI in the UAs and UmAs of pregnant female progeny from DG- or vehicle-treated dams (Figure 5B,C,E,F). Figure 5A,D display the location of the UA and UmA in situ, respectively, as well as a representative Doppler flow diagram. In line with our previous observations in the dams, DG did not affect the maternal-fetal circulation in subsequent pregnancies of the female progeny.

Early-pregnancy DG treatment of pregnant dams provoked an increase in uterine *FOXP3* mRNA expression and in uterine IL-10-expressing CD4+ T cell proportions in pregnant female progeny.

Finally, we checked whether previous DG treatment of pregnant dams would affect the uterine immune composition in next-generation pregnancies and may therefore possess long-lasting effects on the local environment. Our data revealed a significant augmentation in the uterine *FOXP3* mRNA expression (Figure S3D) and in the proportions of uterine IL-10-expressing CD4+ T cells (Table 3) in pregnant female progeny of dams that had been treated with both DG doses or the 2 mg/kg DG dose, respectively. All other immune parameters studied were not altered by previous DG treatment (Figure S3 and Table 3). Previous in utero exposure to DG resulted in minor changes in the uterine immune compartment of pregnant female progeny at midgestation.

## 3. Discussion

Progestogens are frequently administered to ART patients as luteal-phase support to improve clinical pregnancy rates, and to patients with threatened and recurrent miscarriages to anticipate early pregnancy termination. Several application routes for progestogen treatments are described. Most of them are associated with certain adverse effects such as pain at injection sites, inflammatory responses and local abscesses in the case of intramuscular application or vaginal irritation, discharge, bleeding, and interference with coitus in the case of vaginal application. DG as an oral application form shows a lower frequency of adverse effects, and thus has a higher level of patient compliance [10]. Moreover, DG has proven itself as a drug with good bioavailability, and exhibits comparable efficacy to other progestogens in terms of clinical pregnancy rates and live birth rates [4,5]. However, data regarding the safety and tolerability of DG are still limited, and there is a knowledge gap regarding potential long-term consequences for the progeny after DG exposure in utero.

In our current study, we evaluated the immediate effects of early-pregnancy DG administration on intrauterine placental and fetal development, maternal-fetal circulation, and immune modulation, as well as DG-driven long-term effects on progeny’s growth and their reproductive performance. We performed our analyses in the mouse system applying DG via the oral route, for the duration of the ‘first trimester’ and in doses comparable to ART patients.

First, we sought to define whether DG, if present during the preimplantation period, may change the molecular and cellular composition of the uterine environment as a critical feature for an adequate embryo implantation. We observed that preimplantation DG treatment in the pregnant mouse provoked only minor changes in the uterine immune compartment without a clear preference for an induction of a more anti- or pro-inflammatory environment. It rather seemed that DG affected specific immunological parameters maintaining the delicate balance of pro- and anti-inflammatory immune responses required for proper embryo implantation. Moreover, DG did not impact on molecules reported to contribute to embryo implantation and placentation by inducing angiogenesis (vascular endothelial growth factor (VEGF)), degrading extracellular matrix components, and promoting tissue remodeling processes (matrix metalloproteinase 9 (MMP9), urokinase-type plasminogen activator (uPA)) [11,12]. However, another study showed that DG is able to up-regulate galectin-1 expression in mice [13], a molecule highly implicated in endometrial receptivity, placental angiogenesis, and maternal-fetal immune tolerance [14]. In our present study, DG did not increase or decrease the likelihood of the mouse embryo to implant, which was in agreement with two older human studies from the 1980s and 1990s showing no significant differences in pregnancy rates between DG and placebo-treated ART patients [15,16]. In contrast, a more recent Cochrane analysis provided evidence that progestogen supplementation during the luteal phase is associated with higher rates of ongoing pregnancies and live births than placebo supplementation; and this was independent of the application route [6].

Second, we wondered whether DG may affect other critical pregnancy-related processes such as fetal and placental growth, fetal-maternal circulation, fetal immune tolerance, and fetal survival. Follow-up analyses of intrauterine growth of whole implantation sites from GD5 to 12 revealed larger implantation sites in DG-treated pregnant dams as compared to vehicle-treated pregnant dams at GD10. However, the observed DG-driven differences in implantation sizes were only visible short-term and were not reflected by differences in fetal and placental parameters at GD12. Moreover, DG application did not interfere with fetal survival as indicated by comparable abortion rates among the groups. This finding was in agreement with observations made in ART patients who did not show enhanced miscarriage rates after DG administration when compared to vaginal progestogen administration [17,18], and in the prevention of threatened and recurrent miscarriages, DG administration was associated with even better outcomes [19,20,21,22]. This might be partly explained by an improved endometrial blood flow in miscarriage patients after DG administration [23]. However, this was not confirmed in another human study [24], and was also not visible in our pregnant mice, in which neither maternal nor fetal blood flow parameters were altered. P4, in general, possesses a variety of immune-modulating properties [1]; however, studies focusing on immune regulation by DG are scarce. Our own analyses of immune parameters at GD12 after DG supplementation showed no changes in proportions of B and T cell subsets and associated cytokines, with the exception of a decrease in uterine *IL-10* expression and a decrease in the number of IL-10- and PD-1-expressing CD4+ T cells. This suggests a decline in anti-inflammatory immune responses, but without any consequences for the maintenance of fetal tolerance. Previous in vitro studies using human material supported the idea that DG downregulates the secretion of Th1- and Th17-related cytokines and upregulates Th2-related cytokines [25,26]. Another study, however, found no influence of DG on in vivo levels of Th1 and Th2 cytokines [27], and it can be speculated that DG affects immune cells differentially in vitro and in vivo.

Third, we were interested in studying long-term effects that became visible in the progeny of DG-exposed pregnant dams. We focused our analyses on the weight gain of newborns up to adulthood and assessed the reproductive potential of the progeny. Remarkably, administration of the high DG dose to pregnant dams resulted in a shift of the female-to-male ratio, with significant more female progeny in this group. Several reasons may account for this observation. DG may induce the phenomenon of sex reversal in mice [28], which means that genotypic XY males become phenotypic females. Consequently, the number of progeny phenotypically defined as females misleadingly increases. We excluded this option, as we confirmed progeny’s phenotypic sex and chromosomal sex to be concordant. Then, DG may preferably provoke the rejection of male fetuses. However, we did not observe elevated abortion rates in the DG-treated dams that would support this assumption. Lastly, DG may particularly increase the fitness of female blastocysts and/or elevate their capability to implant in the maternal endometrium. In this regard, it has been suggested that maternal hormones are able to influence progeny’s sex ratios through developmental asynchrony by altering the preparation of the uterus and the developmental rate of blastocysts [29,30]. An in vitro study provided evidence that the addition of P4 to mouse oocytes during fertilization decreased the developmental progression to the morula stage and favored the development of male blastocysts. However, this contradicts our in vivo findings for DG, and it clearly indicates that P4 in general can affect the progeny’s sex ratio in very early developmental stages [31]. Nevertheless, our own observations have to be confirmed in DG-treated ART patients whose embryos underwent preimplantation genetic testing, which allows, despite exclusion of genetic abnormalities, the determination of the embryo’s sex before its transfer into the uterine cavity. If embryos of both sexes are transferred, a preference for implantation of embryos of one or the other sex under DG treatment can be determined.

Unhealthy weight development during childhood (over and underweight) foreshadows serious health consequences, such as the development of type 2 diabetes, hypertension, cardiovascular diseases, asthma, and an impaired immune functionality with a higher susceptibility to infections [32,33,34,35,36]. To understand whether exposure to DG in utero may increase the risk of the progeny for the development of civilization diseases, we evaluated progeny’s weight development after birth. Weight gain analyses of both sexes revealed no DG-mediated alterations, with the exception of a short-term increase in female progeny’s weight at the age of 5 weeks in the low-dose DG-treated group. Previously, one study suggested an association between first-trimester maternal P4 levels and female birthweight [37]; however, whether increased P4 levels during early pregnancy stages can indeed affect weight development of female progeny remains to be clarified. Our data did not support harmful effects on progeny’s weight development after in utero DG exposure. A recent systematic review pointed out that prenatal P4 administration (second and third trimester) to prevent preterm birth showed no effect on child development; however, the authors identified an urgent need for follow-up studies of early-pregnancy P4 administration and related effects in offspring beyond early childhood [38]. Caution in this respect should also arise from observations that steroid hormones, if applied during pregnancy, might lead to cancer development later in life [8,39]. We addressed a potential influence of DG application during early pregnancy stages on the reproductive performance of the progeny of both sexes. Female progeny of DG-treated dams exhibited normal estrus cycle lengths and comparable corpora lutea numbers, suggesting no reduced capability to achieve pregnancy. Indeed, pregnancy rates of DG-exposed female progeny were comparable to vehicle-exposed female progeny, and no abnormalities in placental and fetal development were discovered. Moreover, maternal and fetal blood-flow indices were unchanged. Analyses of uterine immunological parameters revealed a higher expression of the Treg-specific transcription factor *FOXP3* and an increased proportion of IL-10-producing CD4+ T cells in pregnant female progeny of DG-treated dams. However, these changes in the immune compartment did not affect fetal survival. Our findings suggested that in utero exposure of female progeny to DG seemed to have no detrimental consequences on their own reproductive capacities. By contrast, male progeny of DG-treated dams showed significant reduced total counts of sperms (low DG dose) and reduced sperm viabilities, together with an increased sperm motility (high DG dose). However, as male progeny of DG-treated dams did not show a reduced capability to impregnate female mice, we propose that DG-driven alterations in sperm parameters did not interfere with the overall reproductive capacity of these males. This is supported by a recent study showing no correlation between spermiogram parameters (total sperm count, sperm concentration, sperm motility) and pregnancy probability in ART patients [40].

The sex-specific DG effects that we observed in our mouse model might be explained by one recent study in which the authors described in a clinic realistic ovine model that early-pregnancy P4 supplementation increases male, but not female, fetal P4 concentrations. As a consequence, expression of gonadotrophins and P4 receptors in the pituitary was reduced in male fetuses but in not female fetuses, indicating sex-specific alterations in pituitary gonadotrophin function driven by P4 treatment. Moreover, in fetal testis, P4 supplementation resulted in an altered gene-expression profile in Leydig and Sertoli cells and affected P4 metabolizing pathways. Based on their results, the authors suggested that maternal P4 supplementation had effects on the reproductive axis development/function in early gestation of the male fetus, but not the female fetus [41]. In our mouse model, we may speculate that exogenous DG administration during early pregnancy stages increased male fetus-specific P4 concentrations, interfered with testis development, and impaired testicular function and spermatogenesis postnatally. Follow-up studies are needed to prove whether this is indeed the case, and whether it also applies to humans. Notably, a previous study indicated an association between early-pregnancy DG application and congenital heart malformations [9]; however, these findings were questioned by other researchers due to several weaknesses of the study [42]. Moreover, two other studies reported comparable incidence of congenital familial and genetic disorders between oral DG and vaginal P4 [43,44].

## 4. Materials and Methods

### 4.1. Animals

Wild-type (WT) C57BL/6J and WT BALB/c females, as well as WT BALB/c males, were purchased from Janvier Labs (Le Genest-Saint-Isle, France). All animals were maintained in our animal facility and treated according to the institutional guidelines with the ministerial approval (Landesverwaltungsamt Saxony-Anhalt; approval date of 27 December 2019; AZ42502-2-1596 UniMD). The experiments were conducted by authorized persons according to the Guide for Care and Use of Animals in Agriculture Research and Teaching. Mice were kept under a 12 h light/12 h dark cycle at 22 ± 2 °C and an air humidity of 40–60%. Water and food were provided ad libitum.

### 4.2. Experimental Design and Tissue Sampling

Eight-week-old C57BL/6J females were mated allogeneically to 8–10-week-old BALB/c males and checked twice a day for a vaginal plug, the appearance of which was equalized to day 0 of gestation. Following that, pregnant females were randomly divided and integrated in three different experimental setups (Figure S4). In the first set of experiments, females were treated in the preimplantation period from GD0 to 4, and in the second and third experimental setup, treatment was carried out from GD0 to 10, which matches the first-trimester period in pregnant women. In each experimental setup, pregnant females received either 2 mg/kg DG (Duphaston^®^, Mylan Healthcare, Bad Homburg, Germany) in 200 µL 0.1% ethanol, 6 mg/kg DG in 200 µL 0.1% ethanol, or 200 µL 0.1% ethanol (vehicle; control group) once per day by oral gavage. The higher DG dose of 6 mg/kg body weight per day corresponds to the daily supplementation of pregnant patients during their first trimester. The DG dose of 2 mg/kg body weight was chosen to determine whether lower DG doses may promote similar effects while showing fewer side effects. Both DG doses were calculated according to human-to-mouse dose translation charts published by Nair and Jacob [45]. At GD5 (first experimental setup), pregnant females were sacrificed, and the number of implantations was recorded. Uterine tissue was collected and further processed for real-time PCR (RT-PCR) and flow cytometry (FC) analyses. In pregnant females that were treated until GD10 (second experimental setup), intrauterine fetal and placental development, as well as maternal and fetal blood-flow parameters, were followed-up by high-frequency sonography until GD12. At the same day, these pregnant females were sacrificed, pregnancy outcome was recorded, fetal and placental weights were measured, and the placental-fetal (placental) index (placental weight/fetal weight) was calculated. Uterine tissue was collected for RT-PCR and FC analyses. Some of the pregnant females that were treated until GD10 (third experimental setup) did not undergo sonography, and gave birth between GD18 and 21. On the day of birth, the total number of progeny per dam was recorded and the sex of each progeny was determined based on the different anogenital distance of female and male mice [46]. At 3 weeks of age (weaning), all progeny were separated from their mothers, and female and male progeny were caged separately. At this time, tail tips were taken for genotyping. The sex-specific weight gain of the progeny was followed up once a week from birth to a maximum of 8 weeks of age. At 6 weeks of age, some female and male progeny were sacrificed to perform estrus cycle analyses and to assess sperm parameters. Eight-week-old male progeny were mated to BALB/c females (ratio of 1:1) for a maximum of 7 days to study their capability to copulate and impregnate the females. Presence of a copulation plug was checked twice a day, and pregnancy was confirmed at GD12. Eight-week-old female progeny were either sacrificed immediately to determine the number of corpora lutea in their ovaries or mated to BALB/c males. Pregnant female progeny underwent sonography to follow up fetal and placental development, as well as to evaluate maternal and fetal blood-flow parameters. At GD12, pregnant female progeny were sacrificed, pregnancy outcome was recorded, fetal and placental weights were measured, and the uterine tissue was collected for RT-PCR and FC analyses.

### 4.3. High-Frequency Sonography

In order to follow up intrauterine placental and fetal development, pregnant females underwent ultrasound examination at GD5, 8, 10, and 12 using the Vevo^®^ 2100 System (Fujifilm VisualSonics Inc., Toronto, ON, Canada) as we previously described [47]. Briefly, female mice were anesthetized with isoflurane, fixed in dorsal position, and ventrally depilated by cream application. Depending on day of examination, implantation size (GD5, 8, 10, and 12), placental area, thickness, and diameter (GD10 and 12) were measured. Additionally, peak systolic velocities (PSV) and end-diastolic velocities (EDV) of the UA (GD5, 8, 10, and 12) and UmA (GD12) were recorded and analyzed with the Vevo LAB software. The software automatically calculated the RI (RI = (PSV − EDV)/PSV) and the PI (PI = (PSV − EDV)/velocity time integral).

### 4.4. Pregnancy Outcome Determination and Uterine Tissue Processing

At GD5 and 12, females were sacrificed, the abdomen was opened, and the bicornial uterus was removed. Both uterine horns were opened longitudinally, and the total number of implantation and abortion sites (only GD12) was recorded. Abortion sites were defined as necrotic and hemorrhagic tissue residues, and the abortion rate was calculated as: (number of abortion sites/total number of implantation sites) × 100. Then, fetal placental units were separated from the surrounding uterine tissue, and one piece of the uterus was kept at −80 °C for RT-PCR analysis. The remaining uterine tissue was digested in Roswell Park Memorial Institute (RPMI) 1640 medium (ThermoFisher, Dreieich, Germany) containing 50 µg/mL liberase (Roche, Grenzach-Wyhlen, Germany) and 5% penicillin/streptomycin (P/S, ThermoFisher, Dreieich, Germany) for 30 min in the incubator at 37 °C and 5% CO_2_. Following incubation, liberase activity was stopped by adding RPMI 1640 medium containing 1% P/S and 10% fetal bovine serum (FBS, ThermoFisher, Dreieich, Germany), uterine tissue pieces were forced through a 100µm cell strainer (Corning, Glendale, AZ, USA), and uterine cells were again incubated for 30 min at 37 °C and 5% CO_2_. Afterward, cells were centrifuged, resuspended in RPMI 1640 medium supplemented with 1% P/S and 10% FBS, and stimulated with phorbol 12-myristate-13-acetate (50 ng/mL, Sigma, Steinheim, Germany), ionomycin (500 ng/mL, ThermoFisher, Dreieich, Germany), and brefeldin A (10 μg/mL, BioLegend, Amsterdam, The Netherlands) for 5 h at 37 °C and 5% CO_2_ in the incubator.

### 4.5. Flow Cytometry

After stimulation of uterine cells, extra- and intracellular antibody staining was performed to determine the proportions of B and T cell subsets in the uterus at GD5 and 12. Briefly, cells were suspended in an FC buffer of phosphate-buffered saline (PBS, PAN Biotech, Aidenbach, Germany) containing 1% bovine serum albumin (Merck Millipore, Darmstadt, Germany) and 0.1% sodium azide (Sigma, Steinheim, Germany). Afterward, staining for extracellular markers was performed for 30 min at 4 °C in the dark. Following a washing step in FC buffer, cells were fixed for 1 h at room temperature. Next, cells were washed in permeabilization buffer and incubated for 30 min at room temperature to realize intracellular staining. For this, the Fix/Perm Buffer Set (BioLegend, Amsterdam, The Netherlands) was used. Then, cells underwent another washing step in permeabilization buffer, were suspended in FC buffer, and finally read on the multicolor flow Attune NxT Flow Cytometer (ThermoFisher, Dreieich, Germany). Data analysis was conducted using the Attune NxT software. Primary gates were set on lymphocytes in FSC/SSC plots, followed by exclusion of doublets and dead cells (Figures S5–S7). Secondary, fluorescence minus one (FMO) controls were applied to distinguish between positive and negative cells (Figures S5–S7). The following antibodies were applied: AF 700-labeled antimouse CD45 (clone: 30-F11), FITC-labeled antimouse CD4 (clone: RM4-5), eFlour506-labeled Fixable Viability Dye (FVD), APC-labeled CTLA-4 (clone: UC10-4F10-11), BV421-labeled antimouse PD-1 (clone: J43), PE-Cy7-labeled antimouse ICOS (clone: 7E.17G9), PE-Cy7-labeled antimouse IL-17A (clone: eBio17B7), PE-eFluor 610-labeled antimouse RORgt (clone: B2D), BV412-labeled antimouse IL-10 (clone: JES5-16E3), PE-eFlour610-labeled antimouse FOXP3 (clone: FJK-16S), PerCP-Cy5.5-labeled antimouse TNFa (clone: MP6-XT22), BV421-labeled antimouse IFNg (clone: XMG1.2), and BV605-labeled antimouse CD19 (clone: 1D3). Anti-CD4, -CD19, -PD-1, -CTLA, -IL-10, -IFNg, and -TNFa were purchased from BD Bioscience, Germany, and anti-FOXP3, -ICOS, -RORgt, -IL-17A, -CD45, and -FVD were purchased from eBiosciences, Frankfurt a. Main, Germany.

### 4.6. Real-Time RT-PCR

Frozen uterine tissue was treated with Trizol^®^ Reagent (ThermoFisher, Dreieich, Germany) and disaggregated using a homogenizer (Ultra-Turrax T8; IKA, Staufen im Breisgau, Germany). RNA was extracted with chloroform, precipitated with 2-propanol, washed in 80% ethanol, and finally diluted in RNase-free water. The RNA was quantified by reading ultraviolet absorbance at 260 nm. For cDNA synthesis, 2 μg of total RNA was placed for 2 min on ice and added with dNTPs (2.5 mmol/L, Amersham Biosciences, Freiburg, Germany), DNase I (2 U/mL, Sigma, Steinheim, Germany), and RNase inhibitor (40 U/mL, Promega, Mannheim, Germany) mixed in reaction buffer. The mix was incubated for 30 min at 37 °C and further heated to 75 °C for 5 min. The addition of the reverse transcriptase (200 U/mL, Amersham Biosciences, Germany) and RNase inhibitor started the reverse transcription. This reaction mixture was incubated at 42 °C for 1 h, followed by incubation at 94 °C for 5 min. For detection of *IL-10*, *TGFb*, *IFNg*, *TNFa*, and *FOXP3*, TaqMan technology was conducted, and for detection of *IL-1b*, *IL-6*, *VEGF*, *MMP9*, and *uPA*, SybrGreen was used. In both cases, PCR reactions were run on an iQ5 Multicolor RT-PCR Detection System (Bio-Rad Laboratories, Munich, Germany). The amplification reactions (12 μL) consisted of 1 μL of cDNA; 6.25 μL of mastermix containing PCR buffer, dNTPs, MgCl2, and Ampli-Taq DNA polymerase (ThermoFisher, Dreieich, Germany); 3 μL of the primer mix; 1.25 μL of water; and 0.5 μL of the fluorescent probes (TaqMan) or 0.5 µL of fluorescein (SybrGreen). PCR reaction was performed as follows: 2 min at 50 °C, followed by an initial denaturation step of 10 min at 95 °C, followed by 15 s at 95 °C, and 1 min at the appropriate annealing temperature for 40 cycles. For SybrGreen analyses, PCR reaction was followed by a melting curve analysis for 80 cycles with an increase in temperature by 0.5 °C in each cycle. *β-Actin* was employed as a housekeeping gene, and relative gene expression was calculated by the 2^−ΔCT^ method. Primer and probe sequences are available upon request.

### 4.7. Tissue and Body Weight Determination

After fetal-placental units were separated from their surrounding tissue, the weight of all fetuses and their corresponding placentas was measured with a microscale (Kern & Sohn GmbH, Balingen, Germany). Body weight of the progeny was taken weekly starting at the day of birth for a maximum of 8 weeks for each progeny separately using the same scale.

### 4.8. Genotyping for Sex Determination

Isolation of genomic DNA from tail tips was performed as described previously [48]. Briefly, tails were lysed in lysis buffer containing Tris hydrochloride (100 mM, Sigma, Steinheim, Germany), ethylenediaminetetraacetic acid (5 mM, Calbiochem, Darmstadt, Germany), sodium chloride (200 mM, Merck, Darmstadt, Germany) and sodium dodecyl sulfate (0.2%, Sigma). After centrifugation, isopropanol was added to supernatants in order to precipitate the DNA. Following a second centrifugation step, DNA pellets were dried and resuspended in DNAse-free water. DNA was quantified, and a classical PCR reaction was conducted according to the protocol published by Clapcote and Roder [49]. Afterward, PCR products were loaded onto an agarose gel, and electrophoresis was carried out. Depending on the number of bands displayed on the gel, females (one band) or males (two bands) were identified. A representative gel is shown in Figure S8.

### 4.9. Estrus Cycle Analysis and Corpora Lutea Count

Estrus cycle analyses were undertaken for 12 consecutive days with a 2-day break after 5 days to circumvent entrance into anestrus due to mechanic intervention. Vaginal lavage was performed by flushing the vagina several times with 50 μL sterile PBS. Cycle stage (diestrus, proestrus, estrus, or metestrus) was defined after observation of the cellular composition under a light microscope (Axiovert 40C, Carl Zeiss, Jena, Germany).

Ovaries were collected from 8-week-old female progeny, kept in 4% paraformaldehyde solution, washed two times in PBS, and finally kept in 70% ethanol until paraffin embedding of the tissue was carried out using the Sainte-Marie method. To circumvent counting the same corpus luteum twice, two cut sections were taken from each ovary with an interval of 500 µm. Each cut section had a thickness of 5 µm. Cut sections were deparaffinized and stained for hematoxylin and eosin. Determination of the number of corpora lutea per ovary was conducted as described previously [50].

### 4.10. Sperm Count, Viability and Motility Analyses

Six-week-old male progeny were sacrificed, the abdomen was opened, and the cauda epididymis was removed. After transferring the tissue into PBS, it was cut three times to release the sperms into the fluid, and 30 µL of sperm solution was mixed with 219 µL PBS and 1 µL FVD (diluted 1:200) and incubated for 10 min at room temperature. Following that, 50 µL of CountBright^TM^ Absolute Counting Beads (ThermoFisher, Dreieich, Germany) were added to the mixture. Total sperm number and viability were then assessed via flow cytometry using the multicolor flow Attune NxT Flow Cytometer. Sperm evaluation took place within 30 min after epididymis removal to avoid deleterious effects of pH and temperature changes on sperm viability and motility. Total number of sperms within the sample was calculated using the following formula: sperms per (µL) = (Number of cell events/number of bead events) × (assigned bead count of the lot/volume of sample (µL)). Sperm motility was evaluated via light microscopy, and the number of progressive sperms (spermatozoa moving linearly or in a large circle), nonprogressive sperms (in situ motility without progression), and immobile sperms (no movement) was determined [51].

### 4.11. Data Analysis and Statistics

Data analysis was conducted with GraphPad Prism 8.0 (Statcon, Witzenhausen, Germany) and SPSS Statistics 24 (IBM, Armonk, NY, USA) software. All data sets were analyzed for normal distribution using the Shapiro–Wilk test. Accordingly, data was assessed for statistical differences using parametric or nonparametric tests. The exact test used for each data set is indicated in the respective figure legend. In all cases, *p* ≤ 0.05 was considered to be statistically significant.

## 5. Conclusions

In conclusion, our current findings corroborated the use of oral DG during early pregnancy stages, as we did not identify major safety issues on pregnancy itself, as well as on the growth development and reproductive performance of the progeny. Nevertheless, early-pregnancy P4 supplementation should not be unnecessarily extended, and should be more individualized based on the patient’s characteristics [52].

## Figures and Tables

**Table 1:** Dydrogesterone (DG) treatment in the preimplantation phase did not profoundly change the uterine composition of adaptive immune cells. Pregnant females were injected with 0.1% ethanol (*n* = 8), 2 mg/kg DG (*n* = 9), or 6 mg/kg DG (*n* = 8) from GD0–GD4. Data are shown as means plus S.D. and were analyzed for statistical differences by using one-way ANOVA followed by Tukey’s multiple comparison test. * *p* < 0.05; ** *p* < 0.01 vs. 2 mg/kg DG.

	0.1% Ethanol (Control)	2 mg/kg DG in 0.1% Ethanol	6 mg/kg DG in 0.1% Ethanol
B cell subsets			
CD19+	5.40 ± 2.00	5.37 ± 2.06	5.19 ± 2.33
CD19+IL-17+	0.76 ± 0.63	0.48 ± 0.43	1.13 ± 0.66
CD19+IL-10+	0.26 ± 0.23	0.17 ± 0.17	0.74 ± 0.55 *
T cell subsets			
CD4+	6.48 ± 1.75	5.96 ± 1.94	6.21 ± 1.46
CD4+IL-17+	0.71 ± 0.27	0.98 ± 0.59	1.29 ± 0.67
CD4+RORgt+	0.71 ± 0.49	0.38 ± 0.26	1.63 ± 1.04 **
CD4+TNFa+	0.85 ± 0.50	1.65 ± 0.82	1.30 ± 0.76
CD4+IFNg+	1.72 ± 0.71	2.20 ± 1.78	1.54 ± 1.14
CD4+IL-10+	1.66 ± 1.28	2.00 ± 1.51	1.76 ± 0.99
CD4+FOXP3+	0.78 ± 0.56	1.60 ± 1.41	1.57 ± 0.94
CD4+CTLA-4+	1.13 ± 1.01	1.85 ± 1.01	1.46 ± 0.69
CD4+PD-1+	6.70 ± 2.23	6.41 ± 1.10	7.72 ± 2.55
CD4+ICOS+	1.23 ± 0.98	1.81 ± 0.78	1.93 ± 0.40

**Table 2:** Dydrogesterone (DG) treatment induced a diminution in two regulatory T cell subsets. Pregnant females were injected with 0.1% ethanol (*n* = 8), 2 mg/kg DG (*n* = 9), or 6 mg/kg DG (*n* = 9) from GD0-GD10. Data are shown as means plus S.D. and were analyzed for statistical differences by using one-way ANOVA followed by Tukey’s multiple comparison test. * *p* < 0.05 vs. control.

	0.1% Ethanol (Control)	2 mg/kg DG in 0.1% Ethanol	6 mg/kg DG in 0.1% Ethanol
B cell subsets			
CD19+	6.31 ± 2.09	5.52 ± 1.25	7.14 ± 1.76
CD19+IL-17+	0.09 ± 0.11	0.09 ± 0.07	0.08 ± 0.10
CD19+IL-10+	0.14 ± 0.21	0.18 ± 0.21	0.30 ± 0.34
T cell subsets			
CD4+	8.65 ± 0.37	9.31 ± 1.71	8.38 ± 1.94
CD4+IL-17+	0.57 ± 0.58	0.55 ± 0.30	0.36 ± 0.43
CD4+RORgt+	0.88 ± 1.26	0.81 ± 0.86	0.34 ± 0.38
CD4+TNFa+	0.79 ± 0.35	0.96 ± 0.85	1.09 ± 0.74
CD4+IFNg+	0.35 ± 0.19	0.27 ± 0.31	0.35 ± 0.40
CD4+IL-10+	0.37 ± 0.30	0.08 ± 0.10 *	0.18 ± 0.15
CD4+FOXP3+	0.86 ± 0.88	0.35 ± 0.38	0.57 ± 0.52
CD4+CTLA-4+	1.21 ± 1.22	0.53 ± 0.59	0.81 ± 0.76
CD4+PD-1+	4.61 ± 2.79	2.26 ± 1.32 *	2.35 ± 1.33
CD4+ICOS+	4.27 ± 5.02	1.63 ± 1.98	2.47 ± 2.77

**Table 3:** Dydrogesterone (DG) treatment augmented the uterine proportions of IL-10-producing CD4+ T cells in pregnant female progeny. Female progeny of 0.1% ethanol (*n* = 8), 2 mg/kg DG (*n* = 8), or 6 mg/kg DG (*n* = 8) treated dams were impregnated and analyzed for uterine B and T cell subsets at GD12. Data are shown as means plus S.D. and were analyzed for statistical differences by using one-way ANOVA followed by Tukey’s multiple comparison test. * *p* < 0.05 vs. control.

	0.1% Ethanol (Control)	2 mg/kg DG in 0.1% Ethanol	6 mg/kg DG in 0.1% Ethanol
B cell subsets			
CD19+	6.68 ± 1.53	7.77 ± 2.65	6.09 ± 1.03
CD19+IL-17+	0.17 ± 0.09	0.16 ± 0.13	0.18 ± 0.18
CD19+IL-10+	0.81 ± 0.52	0.75 ± 0.42	0.55 ± 0.28
T cell subsets			
CD4+	33.72 ± 7.91	33.10 ± 4.15	33.51 ± 6.72
CD4+IL-17+	0.22 ± 0.21	0.26 ± 0.13	0.28 ± 0.20
CD4+RORgt+	0.53 ± 0.67	0.59 ± 0.39	0.24 ± 0.23
CD4+TNFa+	0.55 ± 0.24	1.68 ± 1.39	0.96 ± 0.67
CD4+IFNg+	0.34 ± 0.27	0.58 ± 0.36	0.25 ± 0.15
CD4+IL-10+	0.24 ± 0.24	1.05 ± 0.93 *	0.33 ± 0.27
CD4+FOXP3+	2.05 ± 1.48	2.40 ± 0.56	2.45 ± 0.73
CD4+CTLA-4+	6.08 ± 3.91	6.75 ± 1.02	6.51 ± 1.99
CD4+PD-1+	19.78 ± 5.45	17.01 ± 2.39	20.53 ± 5.30
CD4+ICOS+	6.02 ± 4.10	8.76 ± 1.89	4.27 ± 1.99

## Data Availability

Primer sequences and probes are available upon request. Data sets were obtained for placental diameters and thicknesses, showing genotypes of all progeny and corpora.

## Supplementary Materials

The following are available online at https://www.mdpi.com/article/10.3390/ijms22105403/s1, Figure S1, Figure S8.
