# Supplementary material for: Early-Pregnancy Dydrogesterone Supplementation Mimicking Luteal-Phase Support in ART Patients Did Not Provoke Major Reproductive Disorders in Pregnant Mice and Their Progeny
Source: Int J Mol Sci. 2021 May 20;22(10):5403. doi: 10.3390/ijms22105403 (PMC8161261; doi:10.3390/ijms22105403)

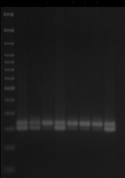

Supplement: Supplementary file 1 [file Figure S8.pdf › Figure S8.pdf]
